# Effect of Auricular Acupressure on Peri- and Early Postmenopausal Women with Anxiety: A Double-Blinded, Randomized, and Controlled Pilot Study

**DOI:** 10.1155/2012/567639

**Published:** 2012-05-10

**Authors:** Ching-Ling Kao, Chao-Hsun Chen, Wei-Yun Lin, Yu-Ching Chiao, Ching-Liang Hsieh

**Affiliations:** ^1^Department of Adult Psychiatry, Tsao-Tun Psychiatric Center and Department of Health, Executive Yuan, Nan-Tou 54249, Taiwan; ^2^College of Chinese Medicine, Graduate institute of Integrated Medicine, China Medical University, Taichung 40402, Taiwan; ^3^Department of Chinese Medicine, Chang-Hua and Department of Health, Hospital, Chang-Hua 51341, Taiwan; ^4^Department of Business Administration, National Chung Hsing University, Taichung 40227, Taiwan; ^5^Graduate Institute of Acupuncture Science, College of Chinese Medicine, China Medical University, Taichung 40402, Taiwan; ^6^Acupuncture Research Center, China Medical University, Taichung 40402, Taiwan; ^7^Department of Chinese Medicine, China Medical University Hospital, Taichung 40402, Taiwan

## Abstract

We tested effects of auricular acupressure on peri- and early postmenopausal women with anxiety (PPWA). Fifty PPWA were randomly assigned to the auricular acupressure group (AG) or the sham group (SG). After 3 meals and before sleep every day for 4 weeks, the AG received auricular acupressure on the bilateral ear shenmen and subcortex points for 3 min per point on alternating ears. The SG received sham auricular acupressure. The Alprazolam was reduced from 0.5 mg/day at baseline to 0.3 mg/day 4 weeks after auricular acupressure (4 W) in the AG (*P* < .05) whereas maintained at 0.5 mg/day in the SG (*P* > .05). The Zolpidem was reduced from 3.0 mg/day at baseline to 1.5 mg/day at 4 W (*P* < .05) whereas was reduced from 2.4 mg/day to 1.9 mg/day at 4 W in the SG (*P* > .05), thus, significant tapering medication, suggesting auricular acupressure is helpful to PPWA.

## 1. Introduction

In menopausal women, anxiety and accompanying insomnia generate a domino effect that can multiply symptoms associated with menopause and depression [1]. These burdens last for more than a few months or even several years, and such conditions often affect quality of life [2]. According to the American Psychiatric Association Diagnostic and Statistical Manual Fourth Edition (DSM-IV, TR) [3], anxiety is classified as an ongoing concern, worry, or sense of impending disaster, a feeling of tension, and an inability to relax. The epidemiology of natural menopause is defined as a lack of menstruation for 12 months in the absence of other causes such as pregnancy or breastfeeding [4]; however, no such standard exists for perimenopause. Perimenopause is characterized by irregular menstrual cycles or less than 12 months without menstruation, but the only index in the first 2 or 3 days of the menstrual cycle is an elevation in the follicle-stimulating hormone (FSH) [5]. A cohort study by a Harvard research team found that significant new depressive symptoms in menopausal women have at least twice the possibility of leading to depression than in the pre-menopausal stage [6]. Anxiety and insomnia can be predictors of depression for women in this period [7]. Anxiety can also predict vasomotor symptoms [8]. An increasing trend of hot flashes, sleep disturbance, physical and mental symptoms, muscle and joint pain, and other symptoms at different stages of menopause may affect women's quality of life [9]. Although estrogen therapy has an effect on vasomotor symptoms, depression, anxiety, and insomnia in menopausal and postmenopausal women [10], the Women's Health Initiative (WHI) reported an increase in cases of coronary heart disease, stroke, venous thrombosis, and dementia cases after hormone therapy [11]. Some studies show that the drugs of choice for insomnia in menopausal and postmenopausal women, such as the nonbenzodiazepines Zolpidem and Eszopiclone, are effective for the short term, and improve sleep, mood, quality of life, and menopause-related symptoms within 4 weeks [12, 13]; however, long-term use of these drugs may still cause psychological or physical dependence.

 Acupuncture reduces anxiety through the regulation of neurotransmitters by reducing the platelet 5-hydroxytryptamine (5-HT) concentration and plasma adrenocorticotrophic hormone (ACTH) [14]. Some randomized controlled studies showed that ear acupuncture before surgery can reduce anxiety related to surgery [15] and can improve anxiety in everyday life for healthy participants [16].

 We used evidence-based methods to review the history and research of the effects of ear acupuncture on anxiety [17–19]. No previous study has explored the therapeutic effect of a combination of auricular acupressure and drug treatment for women suffering from anxiety and insomnia during the menopausal stage; therefore, we designed this protocol in an attempt to provide convenient and effective treatment and to reduce dependence on sedatives, thereby improving quality of life for participants.

## 2. Materials and Methods

### 2.1. Participants

Ninety-one peri- and early postmenopausal women with anxiety (PPWA) were recruited at the Department of Psychiatry, Chang-Hua Hospital, Department of Health, from January 1, 2010 to December 31, 2010. Forty-one patients were excluded from the study prior to signing informed consent: 17 patients were aged more than 65 years old, 12 patients had experienced menopause for more than 10 years, 1 had a serious medical disease, 3 had major psychiatric illnesses, 2 had recently used hormones, 1 regularly used Chinese herbs, and 3 refused the trial. The inclusion criteria were (1) between 40 and 60 years of age; (2) irregular menstruation for less than 12 months or menopause for less than 10 years; (3) FSH plasma concentrations of ≧14 IU/L; (4) anxiety secondary to perimenopause or early post-menopause. Participants provided written informed consent after a full explanation of the purpose and process of the study. Exclusion criteria were (1) serious medical disorders (e.g., asthma, epilepsy, or heart, liver, or renal failure); (2) substance dependence or abuse (e.g., alcohol, drugs, hypnotics, and analgesics); (3) contraindications to sedatives and hypnotic drugs; (4) contraindications to acupuncture treatment; (5) suicidal and violent tendencies; (6) existence of primary anxiety disorders and other major axis I psychiatric diagnoses (e.g., schizophrenia, major depression, and bipolar disorders); (7) lack of fulfillment of the standard type and dosage of the drugs set in this study; (8) use of hormone therapy for menopausal symptoms; (9) use of traditional Chinese medicine for mental conditions; (10) refusal to sign the informed consent form. The plan was reviewed and approved before the trial by the China Medical University Hospital Institutional Review Board (CMUH IRB no. DMR98-IRB-291-1).

### 2.2. Design and Sample Size

The present study is a randomized double-blind control pilot study of a combination of modern medicine and complementary therapy of auricular acupressure to research the change in anxiety symptoms in peri- and early postmenopausal women. Remission of anxiety is defined as a reduction in score on the Hamilton Anxiety Rating Scale (HAMA) [20] ≧50% and Clinical Global Impression-severity/Clinical Global Impression-Improvement/(CGI-S/CGI-I) [21] ≦2, and improvement of menopausal symptoms is defined as a reduction in score on the Menopause Rating Scale (MRS) [22] ≧50%.

Because the present study was a pilot study, no basis existed on which to calculate power or sample size.

### 2.3. Randomized and Grouping

Fifty peri- and early postmenopausal women with anxiety were assigned to 1 of 2 groups by a computerized random numbers table, as follows: (1) in the auricular acupressure group (AG), participants received ear adhesive tape with magnetic beads (200 Gauss, Xiang Yu International Co., Ltd., Taiwan) on the ear shenmen (MA-TF1) and subcortex (MA-AT1) points for both ears from a Chinese medicine physician with more than 8 years of experience in auricular acupressure. Acupressure was applied to each acupoint on alternating ears for 3 min at each point after 3 meals and before bedtime every day for 4 weeks. The ear adhesive tape with magnetic beads was changed twice a week, and the location of magnetic beads was reconfirmed again prior to the changes; thus, they were changed 8 times in the 4 weeks; (2) in the sham acupressure group (SA), the methods were identical to AG, but the ear adhesive tape had no magnetic beads. The checklist of consolidated standards of reporting trials (CONSORT) was complete [23]. The complete details of the intervention are presented in Table 1 in conformance to standards for reporting intervention in controlled of acupuncture [24]. All participants were allowed to receive doses of Alprazolam 0 to 2 mg/day and Zolpidem 0 to 10 mg/day, gradually tapering the dosage of each drug during the period of treatment.

### 2.4. Assessment and Outcome Measure

The assessment was performed by a psychiatric specialist who was blind to the group. The participants were evaluated at baseline (before auricular acupressure) and 4 weeks after auricular acupressure (4W). The type and dosage of medication were also recorded at each visit.

Primary outcome measures focused on the difference in dosage of Alprazolam or Zolpidem between the baseline and at 4 W and the difference in HAMA, MRS, CGI-S, and CGI-I scores between baseline and at 4 W. The secondary outcome measure was the difference in quality of life according to Short Form Health Survey (SF-36) scores and its subscores between baseline and at 4 W.

 The HAMA contains 14 items measured with a 5-point Likert scale. The score is from 0 (no symptoms) to 4 (extremely severe). The present study calculated only the total scores. The higher the total score was, the more serious were the symptoms of anxiety. The MRS includes 11 items that can be divided into 3 subscales: the urogenital, somatic and psychological domains. It uses a 5-point Likert scale from 0 (no symptoms) to 4 (extremely severe). The study calculated only the total scores. The higher the total score, the more serious the symptoms of menopause. The SF-36 contains 36 items to assess physical and mental health, divided into 8 subscales: physical function (PF), social function (SF), role limitations caused by physical problems (RP), role limitations caused by emotional problems (RE), mental health (MH), energy/vitality (VT), body pain (BP), and general perception of health (GH). This study assessed each subscale; higher scores presented a better health status. The CGI-S contains 7 items from 1 to 7 points. A higher score indicates greater severity. The CGI-I contains 7 items from 1 to 7 points. A lower score represents a higher degree of improvement.

### 2.5. Statistical Analysis

 The data were analyzed using statistical software SPSS 18.0 version. The categorical data was analyzed using Pearson x2 tests or Fisher's exact tests. Independent *t*-tests (two-tailed) were used to analyze the variables between the AG and SG groups, and paired *t*-tests (two-tailed) were used to analyze the intragroup variables. Significance of statistical difference was set to *P* < .05.

## 3. Results

### 3.1. Baseline Characteristics of Demographic Data

**Figure 1:**
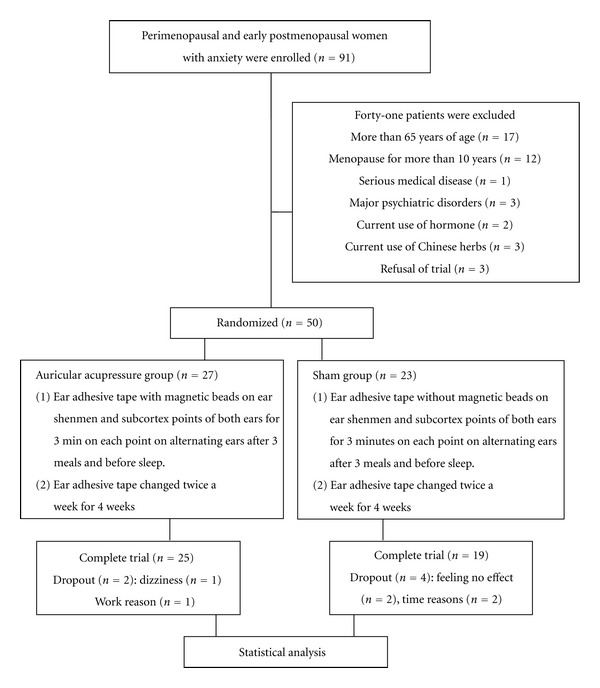
Flowchart.

Of the 50 PPWA enrolled in the present study, 27 participants were assigned to the AG and 23 to the SG. Only 25 participants in the AG completed the trial. One stopped because of dizziness, and 1 stopped because of other reasons. Nineteen participants completed the trial in the SG; 2 stopped because they felt no effect, and 2 could not adhere to the times in the study. Therefore, 44 participants completed the trial (Figure 1). The baseline characteristics of the AG and SG participants, which comprised age, education, parity, age at menarche, marital status, menopausal status (perimenopause and post-menopause), and levels of FSH, were similar between the 2 groups (all *P* > .05; Table 2). In addition, factors influencing anxiety investigated prior to the trial, namely, use of herbal medicine, smoking habits, alcohol habits, caffeine habits, and Brief Symptom Rating Scale (BSRS), were similar between the AG and the SG (all *P* > .05; Table 3). The exception was that prior use of FSH was greater in the SG than in the AG (*P* = .03; Table 3).

### 3.2. Effect of Auricular Acupressure on PPWA

The HAMA scores at baseline and at 4 W were similar between the AG and the SG (both *P* > .05; Table 4). The HAMA scores were higher at baseline than at 4 W in the AG and in the SG (both *P* < .05; Table 4), and the difference in HAMA score between baseline and 4 W was similar between the 2 groups (*P* > .05; Table 4).

 The MRS scores at baseline and at 4 W were similar for the AG and the SG (both *P* > .05; Table 4). The MRS scores were higher at baseline than at 4 W in the AG and in the SG (both *P* < .05; Table 4), and the difference in MRS score between baseline and 4 W was similar for the 2 groups (*P* > .05; Table 4).

 The CGI-S scores at baseline and at 4 W were similar for the AG and the SG (both *P* > .05; Table 4). The CGI-S scores were higher at baseline than at 4 W in the AG and in the SG (both *P* < .05; Table 4), and the difference between CGI-S score at baseline and at 4 W was similar for the 2 groups (*P* > .05; Table 4).

The CGI-I scores at baseline and at 4 W were similar for the AG and the SG (both *P* > .05; Table 4). The CGI-I scores were higher at baseline than at 4 W in the AG and in the SG (both *P* < .05; Table 4), and the difference between CGI-I score at baseline and at 4 W was similar for the 2 groups (*P* > .05; Table 4).

The Alprazolam doses at baseline and at 4 W were similar for the AG and the SG (both *P* > .05; Table 4). The Alprazolam doses were greater at baseline than at 4 W in the AG (both *P* < .05; Table 4), but not in the SG (*P* > .05; Table 4). The difference in Alprazolam doses between baseline and 4 W was similar for the 2 groups (*P* > .05; Table 4).

 The Zolpidem doses at baseline and at 4 W were similar for the AG and the SG (both *P* > .05; Table 4). The Zolpidem doses were higher at baseline than at 4 W in the AG (both *P* < .05; Table 4), but not in the SG (*P* > .05; Table 4). The difference between Zolpidem doses at baseline and at 4 W was similar for the 2 groups (*P* > .05; Table 4).

### 3.3. Effect of Auricular Acupressure on Subscores of SF-36 in PPWA

 The PF sub-score of SF-36 at baseline and at 4 W were similar for the AG and the SG (both *P* > .05; Table 5). The PF subscores of SF-36 were higher at 4 W than at baseline in the AG (both *P* < .05; Table 5), but not in the SG (*P* > .05; Table 5). The difference between PF subscores of SF-36 at baseline and at 4 W was similar for the 2 groups (*P* > .05; Table 5).

 The RP and RE subscores of SF-36 at baseline and at 4 W were similar for the AG and the SG (both *P* > .05; Table 5). The RP and RE subscores of SF-36 were greater at 4 W than at baseline in the SG (both *P* < .05; Table 5), but not in the AG (both *P* > .05; Table 5). The difference between the RP and RE subscores of SF-36 at baseline and at 4 W was similar for the 2 groups (both *P* > .05; Table 5).

 The VT, MH, BP, and GH subscores of SF-36 at baseline and at 4 W were similar for the AG and the SG (all *P* > .05; Table 5). The VT, MH, BP, and GH subscores of SF-36 were greater at 4 W than at baseline in the AG and in the SG (all *P* < .05; Table 5), whereas the difference between VT, MH, BP, and GH subscores of SF-36 at baseline and at 4 W were similar for the 2 groups (all *P* > .05; Table 5).

 The SF sub-score of SF-36 at baseline and at 4 W were similar for the AG and the SG (both *P* > .05; Table 5). The SF sub-score of SF-36 was similar at baseline and at 4 W in the AG and in the SG (both *P* < .05; Table 5). The difference in SF sub-score of SF-36 between baseline and 4 W was similar for the 2 groups (*P* > .05; Table 5).

## 4. Discussion

 The results of the present study indicate that the difference between HAMA, MRS, CGI-S, and CGI-I scores at baseline and at 4 W was similar for the AG and the SG. The doses of Alprazolam and Zolpidem were reduced from baseline to 4 W in the AG, but not in the SG. Therefore, we suggest that auricular acupressure improved PPWA, although the participants of prior use of hormone therapy was more in the SG than AG with significant difference, because the severity of anxiety was not statistically different at baseline, the effect of hormone on outcome may be minimal. Both Alprazolam and Zolpidem are sedative drugs widely used to treat PPWA or insomnia [12, 13], and HAMA, MRS, CGI-S, and CGI-I scores can be used to evaluate menopausal anxiety [22]. This is the first study showing that auricular pressure with low-dose Alprazolam and Zolpidem improves anxiety symptoms, menopausal symptoms, and quality of life in PPWA. The results also show that the dosage of drugs can be gradually reduced during the course of treatment to help avoid long-term drug use that could induce dependence. The present study was only one participant feeling dizziness in the AG, but none in the SG; therefore, the treatment was safe and the side effect was minimal. However, participants in the AG and the SG could not predict which group would show significant improvements. The results are consistent with those of previous studies that found auricular pressure effective for treating anxiety [25] and anxiety before surgery [26] and for improving quality of life [27]. Evidence-based studies on the efficacy of acupuncture have compared the efficacy of real and control acupuncture for clinical conditions such as postmenopausal vasomotor symptoms in women [28], including primary dysmenorrhea [29], insomnia [30], and weight loss [31], finding improvement in real and control acupuncture and showing no statistical difference between the 2 groups. However, most of the studies did not design a treatment group without intervention as a control, instead using a noninvasive method as the control group; therefore, the placebo effect may have contributed to their results. Some scholars have indicated that sham acupuncture does not exist because true acupuncture and sham acupuncture could have similar effects on the central nervous or endocrine systems [32, 33].

 The choice of the ear shenmen and subcortex acupoints was based on the meridian theory of traditional Chinese medicine with a sedative mechanism to regulate cortical excitation and inhibit brain function. Auricular acupressure on these acupoints may alleviate the stimulating effect of anxiety, which promotes blood circulation through the nerve channels. It may also stimulate the small myelinated nerve in the spinal cord, midbrain, pituitary, and hypothalamus, causing the release of endorphins into the bloodstream [34].

 At the end of this study, the average Alprazolam dosage in the AG was 0.3 mg/day and in the SG was 0.5 mg/day. No statistically significant difference between the groups was reached; however, in the AG, the difference in drug use between the beginning and end of the study reached statistical significance. A previous study showed that 1.5 to 3 mg of Alprazolam per day can improve moderate to severe anxiety and has antidepressant effects [35]. Our participants entered the study with mild to moderate anxiety. However, the average dosage remains lower than the general therapeutic dose in clinical use. Further research is required to adjust the experimental design using a fixed dosage of combination drugs to distinguish the difference between the 2 groups.

 The present study has several limitations: (1) The sample size was small. With the first error set to 0.05 and the second error set to 0.2, the power of this study was 0.9; for the study to detect a significant difference in therapeutic effect, each group requires at least 30 participants; (2) the participants are all from a hospital in a rural area, and, therefore, the results may not be representative of other areas; (3) if the participants were interested in auricular acupressure or held a positive view of it before the study, this was likely to affect the results of the study; (4) we had no untreated group as a control. As mentioned, sham acupuncture had a similar effect, but a design considering this cannot be a double-blind experimental design. Previous studies did not directly compare the efficacy of auricular acupressure with different methods such as rapeseed, Vaccaria son, mustard seed, and magnetic beads in reducing anxiety; therefore, future studies might consider them; (5) the therapeutic relationship may provide a feeling of relaxation, causing participants to feel less anxiety or less distressed, consciously or unconsciously driven by the hope of achieving clinical improvement; (6) the timeframe for our study is only 4 weeks. We cannot predict whether the therapeutic effect would be maintained over a longer period; (7) the magnet beads displaced from the acupoint may result in the similar effect as the sham group. In the future, we could design a study that, after using a combination of drugs and auricular pressure for 4 weeks, uses auricular acupressure alone as treatment for a set time. This could avoid drug dependency and assess whether auricular acupressure alone can maintain beneficial effects.

 In conclusion, no direct support was found for our hypothesis that auricular acupressure is better than sham acupressure for improving anxiety, menopausal symptoms, and quality of life. Participants improved whether receiving real or sham acupressure. The dosage of medication decreased significantly in the auricular acupressure group, but not the sham acupressure group. This may be indirect evidence of the benefit of such alternative therapy. Research to develop more safe and effective interventions using integrated complementary therapy and Western treatment should be encouraged. Further study with a larger sample size is necessary.

## Supplementary Material

CONSORT 2010 checklist of information to include when reporting a randomised trial.

## Figures and Tables

**Table 1:** Standards for reporting interventions in clinical trials of acupuncture (STRICTA 2010).

Acupoint rationale	(1) According to meridian theory of traditional Chinese medicine. (2) MA-TF1 (ear shenmen), MA-AT1 (ear subcortex).

Acupressure detail	(1) Bilateral ear acupoints. (2) Four magnetic beads. (3) Magnetic beads on the skin surface in acupressure group (AG), and without magnetic beads in sham group. (4) Pressure feeling. (5) Alternating ears for 3 min at each point. (6) Magnet bead, 200 Gauss, Xiang Yu international Co., Ltd., Taiwan.

Treatment regimen	(1) Four times (after 3 meals and before bedtime) every day for 4 weeks.

Cointerventions	(1) None of herbs, moxibustion, cupping, massage, exercise, advice dietary or lifestyle modification.

Practitioner background	(1) License-certificated Chinese medical doctor with more than eight years of acupuncture experience.

Control intervention	(1) Nil.

**Table 2:** Demographic characteristics at baseline.

	AG (*n* = 25)	SG (*n* = 19)	*P* value
Age (years)	53.56 ± 4.63	54.42 ± 6.42	.607^t^
Education (years)	8.92 ± 3.71	9.47 ± 4.58	.660^t^
Parity	2.71 ± 1.31	3.12 ± 1.36	.360^t^
Menarche (years)	14.25 ± 1.77	13.67 ± 1.24	.267^t^
Marital status			.179^c^
Married	24 (96%)	17 (89.5%)	
Single	1 (4%)	0 (0.0%)	
Divorced	0 (0.0%)	2 (10.5%)	
Menopausal status			.986^c^
Perimenopause	5 (20.9%)	4 (21.1%)	
Post-menopause	19 (79.1%)	15 (78.9%)	
FSH	58.7 ± 28.2	66.2 ± 35.6	.45^t^

Data are expressed as mean ± standard deviation (SD). AG: auricular acupressure group; SG: sham group; FSH: follicle stimulating hormone;

^t^: Student's *t*-test; ^c^: Pearson chi-square or Fisher's exact test.

**Table 3:** Influencing factors at baseline.

	AG (*n* = 25)	SG (*n* = 19)	*P* value
Prior use of hormone	9 (36%)	13 (68.4%)	.03
Herbal medicine	11 (44%)	4 (21%)	.11
Smoking habit	1 (4%)	0 (0%)	.38
Alcohol habit	1 (4%)	0 (0%)	.38
Caffeine habit	6 (24%)	2 (11%)	.23
BSRS	7.6 ± 4.1	8.0 ± 3.2	.76^t^

AG: auricular acupressure group; SG: sham group; BSRS: Brief Symptom Rating Scale. ^t^: Student's *t*-test.

**Table 4:** Effect of auricular acupressure on peri-menopausal and early postmenopausal women with anxiety.

	AG (*n* = 25)		SG (*n* = 19)	
	Baseline	4 W	Baseline	4 W
HAMA	17.8 (6.2)^a^	8.0 (5.5)^a,b^	19.6 (5.5)	11.0 (6.3)^b^
MRS	11.8 (5.9)^a^	6.4 (5.8)^a,b^	14.5 (6.1)	8.2 (4.9)^b^
CGI-S	3.8 (0.7)^a^	2.3 (0.9)^a,b^	4.1 (0.7)	2.7 (1.0)^b^
CGI-I	4.4 (0.5)^a^	2.0 (0.9)^a,b^	4.5 (0.5)	2.6 (1.1)^b^
Alprazolam (mg/day)	0.5 (0.4)^a^	0.3 (0.3)^a,b^	0.5 (0.6)	0.5 (0.6)^c^
Zolpidem (mg/day)	3.0 (4.0)^a^	1.5 (3.1)^a,b^	2.4 (2.6)	1.9 (3.4)^c^

AG: auricular acupressure group; SG: sham group; Baseline: prior to auricular acupressure; 4 W: at 4 week after auricular acupressure; HAMA: Hamilton Anxiety Rating Scale; MRS: Menopause Rating Scale; CGI-S: Clinical Global Impression-Severity; CGI-I: Clinical Global Impression-Improvement; ^a^No significant difference between AG and SG groups (*P* > .05). ^b^Significant difference between baseline and 4 W (*P* ≤ .05). ^c^No significant difference between baseline and 4 W (*P* > .05).

**Table 5:** Effect of auricular acupressure on subscores of SF-36 in peri-menopausal and early postmenopausal women with anxiety.

	AG (*n* = 25)		SG (*n* = 19)	
	Baseline	4 Ws	Baseline	4 W
PF	80.2 (15.0)^a^	87.8 (14.9)^a,b^	73.4 (21.9)	79.7 (20.8)^c^
RP	59.0 (42.6)^a^	75.0 (36.1)^a,c^	46.1 (47.3)	75.0 (37.3)^b^
RE	52.0 (44.2)^a^	68.0 (41.4)^a,c^	49.1 (43.6)	82.5 (32.1)^b^
VT	54.6 (22.4)^a^	65.2 (19.6)^a,b^	49.2 (20.6)	61.3 (20.7)^b^
MH	54.4 (18.4)^a^	65.3 (19.4)^a,b^	50.7 (15.5)	64.4 (16.6)^b^
SF	73.0 (19.3)^a^	79.0 (17.2)^a,c^	68.4 (19.7)	76.3 (16.6)^c^
BP	69.1 (15.7)^a^	76.7 (13.5)^a,b^	60.7 (14.2)	73.8 (19.7)^b^
GH	45.8 (16.8)^a^	62.2 (22.1)^a,b^	36.6 (19.6)	55.8 (23.2)^b^

AG: auricular acupressure group; SG: sham group; Baseline: prior to auricular acupressure; 4 W: at 4 week after auricular acupressure; SF-36: Short Form Health Survey; PF: physical function; SF: social function; RP: role limitations due to physical problems; RE: role limitations due to emotional problems; MH: mental health; VT: energy/vitality; BP: body pain; GH: general perception of health; ^a^No significant difference between AG and SG groups (*P* > .05). ^b^Significant difference between baseline and 4 W (*P* ≤ .05). ^c^No significant difference between baseline and 4 W (*P* > .05).
